# Effect of glucagon like peptide-1 receptor agonist exenatide, used as an intracranial pressure lowering agent, on cognition in Idiopathic Intracranial Hypertension

**DOI:** 10.1038/s41433-023-02908-y

**Published:** 2024-01-11

**Authors:** Olivia Grech, James L. Mitchell, Hannah S. Lyons, Andreas Yiangou, Mark Thaller, Georgios Tsermoulas, Kristian Brock, Susan P. Mollan, Alexandra J. Sinclair

**Affiliations:** 1 Institute of Metabolism and Systems Research, College of Medical and Dental Sciences, University of Birmingham, Birmingham, B15 2TT UK; 2 Centre for Endocrinology, Diabetes and Metabolism, Birmingham Health Partners, Birmingham, B15 2TH UK; 3 Department of Neurology, Queen Elizabeth Hospital, University Hospitals Birmingham NHS Foundation Trust, Birmingham, B15 2WB UK; 4 Department of Neurosurgery, Queen Elizabeth Hospital, University Hospitals Birmingham NHS Foundation Trust, Birmingham, B15 2WB UK; 5 Cancer Research UK Clinical Trials Unit, University of Birmingham, Birmingham, B15 2TT UK; 6 Birmingham Neuro-Ophthalmology, Queen Elizabeth Hospital, University Hospitals Birmingham NHS Foundation Trust, Birmingham, B15 2WB UK

**Keywords:** Cognitive neuroscience, Neurological disorders, Diseases of the nervous system

## Abstract

**Background:**

Cognitive function can be affected in conditions with raised intracranial pressure (ICP) such as idiopathic intracranial hypertension (IIH). Drugs used off label to treat raised ICP also have cognitive side effects, underscoring the unmet need for effective therapeutics which reduce ICP without worsening cognition. The Glucagon Like Peptide-1 (GLP-1) receptor agonist, exenatide, has been shown to significantly reduce ICP in IIH, therefore this study aimed to determine the effects of exenatide on cognition in IIH.

**Methods:**

This was an exploratory study of the IIH:Pressure trial (ISTCRN 12678718). Women with IIH and telemetric ICP monitors (n = 15) were treated with exenatide (n = 7) or placebo (n = 8) for 12 weeks. Cognitive function was tested using the National Institute of Health Toolbox Cognitive Battery at baseline and 12 weeks.

**Results:**

Cognitive performance was impaired in fluid intelligence ((T-score of 50 = population mean), mean (SD) 37.20 (9.87)), attention (33.93 (7.15)) and executive function (38.07 (14.61)). After 12-weeks there was no evidence that exenatide compromised cognition (no differences between exenatide and placebo). Cognition improved in exenatide treated patients in fluid intelligence (baseline 38.4 (8.2), 12 weeks 52.9 (6.6), *p* = 0.0005), processing speed (baseline 43.7 (9.4), 12 weeks 58.4 (10.4), *p* = 0.0058) and episodic memory (baseline 49.4 (5.3), 12 weeks 62.1 (13.2), *p* = 0.0315).

**Conclusions:**

In patients with raised ICP due to IIH, exenatide, a drug emerging as an ICP lowering agent, does not adversely impact cognition. This is encouraging and has potential to be relevant when considering prescribing choices to lower ICP.

## Introduction

Raised intracranial pressure (ICP) is a characteristic of numerous neurological disorders including traumatic brain injury (TBI) [1], stroke [2], subarachnoid haemorrhage [3], hydrocephalus and idiopathic intracranial hypertension (IIH) [4]. Severe headache, visual disturbances and pulsatile tinnitus are recognised features of raised ICP [5], however impairments in cognitive function are also well documented. A recent prospective case-control study in IIH highlighted deficits in executive function and attention, which were reversible with reductions of ICP [6]. Cognitive studies in patients with TBI and hydrocephalus have also identified impairments in problem-solving [7], executive function [8], short-term and working memory [9, 10].

There are currently no licensed treatments dedicated to reducing ICP. There are a number of existing drugs which are used off label to reduce ICP. A recent open label trial compared the efficacy of drugs currently used to lower ICP in IIH (amiloride, furosemide, spironolactone and topiramate) and found a marginal reduction in ICP with no difference between drugs [11]. Of concern, however, was that acetazolamide, spironolactone and topiramate were demonstrated to have worsened cognitive function in participants [12]. Therefore there is an unmet need for therapeutics which can result in a clinically meaningful reduction in ICP without side effects which compromise cognition.

Exenatide, a glucagon like peptide- 1 (GLP-1) receptor agonist, is an existing therapeutic agent used in the treatment of diabetes which has additional weight loss effects [13–15]. Exenatide has been recently shown to reduce cerebrospinal fluid (CSF) secretion and ICP [16]. Exenatide targets the GLP-1 receptors located at the choroid plexus epithelium, the structure responsible for the majority of CSF secretion in the brain [16, 17]. In vivo investigation demonstrated that GLP-1 receptor agonism with exenatide was able to reduce CSF secretion and ICP by at least 45% [16]. The in vivo response represented a greater reduction in ICP than that noted with other drugs commonly used in IIH [18]. A phase 2 randomised placebo controlled double-blind trial (IIH:Pressure trial) demonstrated the efficacy of exenatide at significantly reducing ICP, as measured using highly accurate telemetric ICP monitors, in people with IIH [19].

A number of commonly used off-label ICP lowering drugs, including acetazolamide and topiramate, have a deleterious effect on cognitive function [12, 20, 21]. The impact of exenatide on cognitive function in patients with raised ICP has not been explored. This is a pre-specified nested study of the IIH:Pressure trial that aimed to determine the effects of exenatide on cognition in a cohort of IIH patients with raised ICP.

## Methods

### Trial design

This cognitive evaluation was an exploratory study of the IIH:Pressure trial. The IIH:Pressure trial was a prospective, randomised, parallel group, placebo-controlled trial to evaluate the effects of GLP-1 receptor agonist exenatide on ICP. The study was approved by the West Midlands—Solihull Research Ethics Committee (17/WM/0179) and all subjects provided written informed consent according to Declaration of Helsinki principles. The trial was registered with ISTCRN (12678718). Women with active IIH were identified and recruited from a single tertiary referral hospital (Queen Elizabeth Hospital, University Hospitals Birmingham NHS Foundation Trust, United Kingdom). The results of the main trial have been previously published [19].

### Participants

Women aged between 18 to 60 years who met the diagnostic criteria for IIH were recruited [22]. This included papilledema, a normal neurologic examination except for cranial nerve abnormalities, normal brain imaging without evidence of hydrocephalus or structural lesions, normal CSF composition, and elevated lumbar puncture opening pressure. All had normal brain imaging (apart from radiological signs of raised ICP), and this included magnetic resonance venography or computed tomography venography to exclude venous sinus thrombosis. All eligible patients had optic nerve head swelling in at least one eye and ICP >25cmH_2_0. Those with significant medical co-morbidities, prior CSF diversion procedures, neurovascular stenting or optic nerve sheath fenestration, those currently using GLP-1 receptor agonists, dipeptidyl peptidase-4 (DPP-4) inhibitors or taking drugs that could reduce ICP were excluded. Those taking drugs that might influence ICP discontinued these at least a month prior to enrolment. Patients who were pregnant or those planning pregnancy were excluded, with urine human chorionic gonadotropin (HCG) checked at each study visit. Detailed enrolment criteria are provided in the Supplementary Table 1. All had telemetric ICP monitors (Raumedic^TM^, Germany) which were surgically implanted prior to the baseline visit. Of note, this approach to ICP monitoring was chosen by the patient advisory group due to the negative impact reported by repeated lumbar punctures [23, 24], and the use within the trial was carefully discussed with the individual participants during the consent process.

### Randomisation and Study Treatment

Participants were randomised in a 1:1 ratio to either active treatment with exenatide (Byetta) or placebo using a computer-generated randomisation list compiled by the Birmingham Clinical Trials Unit. Treatment allocation was masked from the participant and investigators and a double check of allocation was performed by an unblinded nurse and pharmacist. The first dose was a loading dose of subcutaneous exenatide 20 mcg or equivalent volume of subcutaneous 0.9% saline placebo. Participants were dosed for 12 weeks (self-administered at home) with either subcutaneous exenatide 10 mcg or equivalent volume of placebo twice daily.

### Cognitive tests

Cognitive testing was performed using the validated National Institute of Health (NIH) Toolbox Cognitive Battery (version 1.11) [25, 26]. The cognitive battery consisted of seven standardised testing paradigms which assess different cognitive domains: crystalised intelligence (relates to knowledge and experience) [27], auditory and oral language, fluid intelligence (abilities to process and integrate) [27], processing speed, working and episodic memory, and attention and executive function. It utilises a computer adaptive testing paradigm allowing assessments to be completed in 40 minutes. Scores are expressed as T-scores (a score of 50 is population mean and a score of +/−10 is one standard deviation from the mean). Individual scores are corrected for age, gender, educational attainment, and ethnicity [28]. The cognitive battery was administered by a trained team member in a controlled, quiet environment under standard lighting conditions (defined as well-lit, neutral illumination devoid of harsh shadows or glare). Testing was performed at baseline and after 12 weeks of exenatide or placebo administration.

### Statistical analyses

Statistical analysis of the cognitive tests was performed using Prism (Prism 8 for MacOS, Graphpad, LCC, Version 8.4.0 (455)). This was an exploratory analysis of the IIH:Pressure trial, the power calculation has been published [19]. Analysis was by intention-to-treat. The normality of data were assessed using quantile-quantile plots and the Shapiro-Wilk test. Baseline cognitive scores were evaluated compared to population normal ranges. Baseline and 12-month cognitive testing in both exenatide and placebo groups were compared using two-tailed *t*-tests. For comparisons between placebo and exenatide two-tailed unpaired *t* tests were used. Statistical significance was considered at *P*  <  0.05 level (two-tailed). There was no missing cognitive data at baseline or 12 weeks.

### Statement of ethics

The study was approved by the West Midlands - Solihull Research Ethics Committee (17/WM/0179) and all subjects provided written informed consent according to the Declaration of Helsinki principles. Participants were screened between 1 November 2017 and 17 September 2018. The trial was registered with ISTCRN (12678718).

## Results

### Patient characteristics

All 15 participants were female, and randomly assigned to receive either exenatide (n = 7) or placebo (n = 8). Exenatide and placebo groups were matched for age (mean (standard deviation (SD) age of exenatide group 28 (13) years, placebo group 28 (6) years) and body mass index (BMI) (exenatide group 37.6 (7.9) kg/m^2^, placebo group 38.6 (4.7) kg/m^2^). Baseline ICP in the left lateral decubitus lumbar puncture (LP) position was similar between groups (exenatide group 30.7 (6.7) cmCSF, placebo group = 33.5 (5.6) cmCSF). Headache and visual parameters were also comparable between groups (Table 1). All randomised patients completed the 12-week duration of the trial and drug compliance was full. ICP was significantly lower in the exenatide group compared to placebo after 12 weeks (exenatide 21.4 (4.0) cmCSF, placebo 26.0 (4.4) cmCSF *p* = 0.058). There were no ICP lowering agents or addition of headache preventative drugs started during the study.

**Table 1:** Clinical characteristics of study participants at baseline. *BMI*, body mass index; *IQR*, interquartile range; *SD*, standard deviation.

	Exenatide Mean (SD)	Placebo Mean (SD)
Number (n)	7	8
Age (years)	28 (13)	28 (6)
BMI (kg/m^2^)	37.6 (7.9)	38.6 (4.7)
ICP (supine) mmHg	22.3 (3.6)	24.6 (4.1)
ICP (Lateral decubitus position) cmCSF	30.7 (6.7)	33.5 (5.6)
Frisén Grade (worst eye) median (IQR)	2 (1)	2.5 (1)
Monthly headache days	21.6 (5.2)	10.3 (8.5)

### Cognitive performance at baseline

**Fig. 1:**
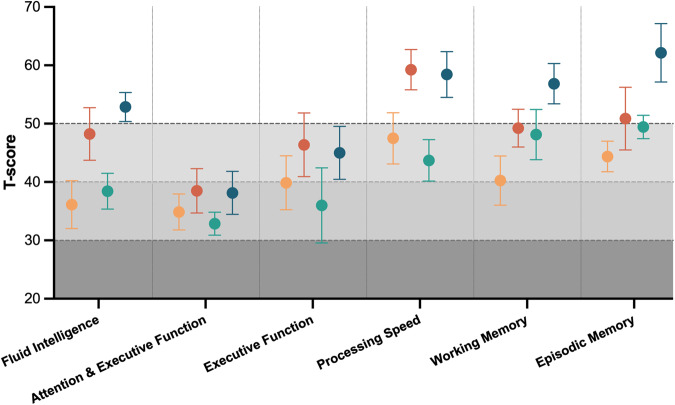
Baseline and 12 week cognitive T-scores in placebo and exenatide groups. a T- score of 50 is considered the population mean and a score of +/−10 is one standard deviation from the mean. Placebo baseline (yellow) 12 weeks (orange), exenatide baseline (green) 12 weeks (blue).

At baseline, performance in numerous cognitive tasks were impaired. These included the fluid intelligence test (T-Score mean (SD) 37.20 (9.87)), flanker inhibitory control and attention test, a test of attention (33.93 (7.15)) and dimensional change card sort test, a test of executive function (38.07 (14.61)). Cognitive domains that were unaffected included crystalized intelligence (51.3 (10.8)) and auditory language (54.8 (14.1)) (Fig. 1). The majority of other cognitive domains were unaffected at baseline (Table 2). Cognitive function was not significantly different between exenatide and placebo groups at baseline.

**Table 2:** T-scores of cognitive domains tested in patients treated with placebo vs exenatide at baseline and 12 weeks. Results expressed as mean (SD). *SD*, standard deviation.

Cognitive domain	All baseline Mean (SD) n = 15	Exenatide Mean (SD) n = 7		Placebo Mean (SD) n = 8	
		Baseline	12-weeks	Baseline	12-weeks
Crystal Intelligence	51.3 (10.8)	53.1 (7.2)	55.6 (12.8)	49.6 (13.6)	56.1 (9.7)
Auditory Language	47.8 (10.4)	46.4 (11.9)	50.3 (12.5)	49.4 (9.1)	48.7 (11.5)
Oral Language	54.8 (14.1)	53.3 (16.6)	60.6 (14.2)	56.6 (11.6)	61.6 (14.3)
Fluid Intelligence	37.2 (9.9)	38.4 (8.2)	52.9 (6.6)	36.1 (11.6)	48.3 (12.7)
Attention & Executive Function	33.9 (7.1)	32.9 (5.2)	38.1 (9.8)	34.9 (8.7)	38.5 (10.8)
Executive Function	38.1 (14.6)	36.0 (17.0)	45.0 (12.0)	39.9 (13.1)	46.4 (15.4)
Processing Speed	45.7 (10.9)	43.7 (9.4)	58.4 (10.4)	47.5 (12.4)	59.3 (9.8)
Working Memory	43.9 (12.0)	48.1 (11.4)	56.9 (9.1)	40.3 (11.9)	49.3 (9.1)
Episodic Memory	46.7 (6.8)	49.4 (5.3)	62.1 (13.2)	44.4 (7.4)	50.9 (15.2)

### Cognitive function following 12-weeks exenatide versus placebo

**Fig. 2:**
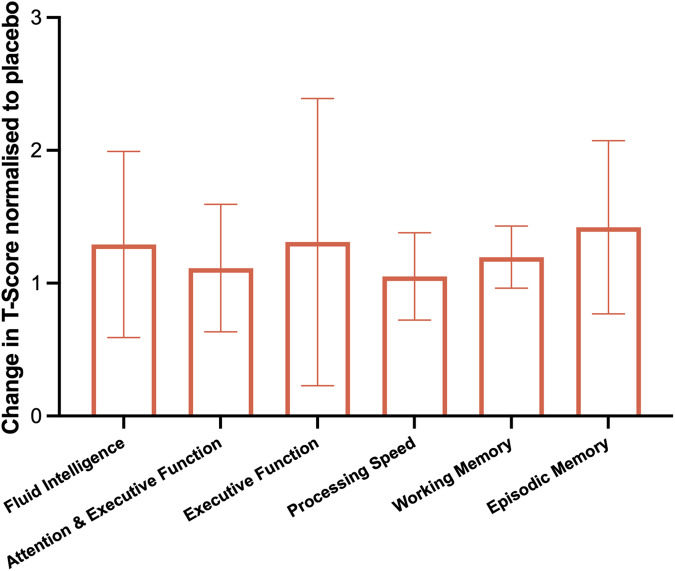
Change in cognitive T-scores in exenatide treated patients normalised to placebo scores. Results expressed as mean change (SD) SD; standard deviation.

The results from the trial describing the ability of exenatide to significantly lower ICP at 12 weeks have been previously published [19]. Following 12-weeks of treatment with the GLP-1 receptor agonist exenatide, cognitive testing was repeated in participants. Cognitive performance in participants receiving exenatide significantly improved in the fluid intelligence domain (T-Score mean (SD) = baseline 38.4 (8.2), 12 weeks 52.9 (6.6), *p* = 0.0005, Fig. 2), processing speed (baseline 43.7 (9.4), 12 weeks 58.4 (10.4), *p* = 0.0058) and episodic memory (baseline 49.4 (5.3), 12 weeks 62.1 (13.2), *p* = 0.0315). Cognition did not significantly worsen in any of the domains.

In participants receiving placebo, there were significant improvements in several domains including crystal intelligence (baseline 49.6 (13.6), 12 weeks 56.1 (9.7), *p* = 0.0457, Fig. 1), fluid intelligence (baseline 36.1 (11.6), 12 weeks 48.3 (12.7), *p* = 0.0001), executive function (baseline 39.93 (13.1), 12 weeks 46.4 (15.4), *p* = 0.0362), processing speed (baseline 47.5 (12.4), 12 weeks 59.3 (9.8), *p* = 0.0025) and working memory (baseline 40.3 (11.9), 12 weeks 49.3 (9.1), *p* = 0.0239). There were no significant differences between the change in cognitive performance in the placebo versus exenatide groups (Fig. 2).

## Discussion

Conditions of raised pressure, such as IIH, are characterised by cognitive impairments which have been shown to be in association with raised ICP [6]. We report baseline cognitive impairments in IIH in fluid intelligence, attention and executive function. This is in agreement with previous assessments of cognition in IIH, where executive function is significantly impaired [6, 29]. In IIH, use of exenatide to reduce ICP did not have a negative impact on cognition at 12 weeks. This is encouraging as many of the existing drugs used to reduce ICP impact on cognitive function [20].

Drugs used commonly in IIH have been shown to worsen cognitive impairments in patients, with acetazolamide and topiramate worsening fluid intelligence [12]. Memory impairment has been reported with acetazolamide use [30], additionally several studies have identified impairments in language, working memory [31, 32], verbal fluency [32], Intelligence quotient, and verbal learning [21]. There is therefore an unmet clinical need for therapeutics which effectively reduce ICP without further impacting on cognitive function.

Exenatide did not enhance cognition compared to placebo in this study, however it did prevent deterioration of cognitive function unlike other drugs used to lower ICP [20]. It is possible that the therapeutic reduction of ICP in the exenatide arm impacted cognitive scores at 12 weeks, as a prior publication showed the beneficial effects of ICP reduction on cognition [6]. Studies have highlighted an association between headache severity and cognitive performance in IIH [6]. Exenatide has demonstrated the ability to headache frequency [19], however its role in headache in raised ICP is yet to be fully determined headaches severity were similar in both trial arms [19]. Of interest is the observation that exenatide exerts neuro-protective effects in animal and clinical studies of Alzheimer’s disease. Exenatide prevented cognitive decline in 5xFAD genetic Alzheimer’s mouse models [33], whist exendin-4 protected against amyloid-β peptide induced impairment of spatial memory and learning in rodents [34, 35]. A reduced incidence of Alzheimer’s disease was found in patients with type-2 diabetes taking exenatide [36], and a 2010 trial was launched to investigate the effects of exenatide on cognitive performance and clinical progression of Alzheimer’s disease [37]. These findings warrants future investigation as to whether exenatide protects cognition via reduction of ICP or other neuroprotective mechanisms.

There are a number of factors and limitations to consider when appraising this study. Although not significant, we did identify improvements in the placebo group which were similar to those treated with exenatide. This may represent regression to the mean or be driven by a learning effect of the cognitive battery. However, we mitigated for this by using variations in the testing paradigms. Although it may be noted that a previous study found no improvement in cognitive function at three months in people with IIH, leading some believe that cognitive deficits in this condition were not reversible [38]. Finally we were not able to control for headache severity which is known to influence cognition in IIH [6], however, this would have impacted both trial arms as headaches scores were analogous in both trial arms at 12 weeks [19].

In conclusion, our findings suggest that exenatide, a drug emerging as an ICP lowering agent in IIH does not adversely impact cognition. A number of drugs prescribed to lower ICP (particularly acetazolamide and topiramate in IIH) compromise cognitive function. This is problematic as patients with raised ICP and IIH have existent clinically relevant cognitive deficits and drug therapy which exacerbates cognitive function further compromises cognition. Our findings have potential to be relevant when considering prescribing choices to lower ICP, since exenatide seemingly avoids further impairment of cognition.

## Summary

### What was known before:

- Cognitive deficits, especially in executive function and attention, have been linked to raised ICP. Various drugs, often used off-label, aim to lower ICP, but some of them negatively affect cognitive abilities. Exenatide, has emerged as a potential solution, however, its impact on cognitive function in individuals with raised ICP remains unexplored.

### What this study adds:

- We also confirm cognitive deficits in IIH patients with raised ICP notably in executive function. The study introduces exenatide as a potential solution for ICP reduction in IIH without negatively impacting cognition. This is significant because the available therapies for lowering ICP often exacerbate existing cognitive deficits.

### Supplementary information

Supplementary Material

## Data Availability

Anonymized individual participant data may be made available along with the trial protocol. Proposals should be made to the corresponding author and will be reviewed by the Birmingham Clinical Trials Unit Data Sharing Committee in discussion with the Chief Investigator. A formal Data Sharing Agreement may be required between respective organisations once release of the data are approved and before data can be released.
