# Supplementary material for: Effect of glucagon like peptide-1 receptor agonist exenatide, used as an intracranial pressure lowering agent, on cognition in Idiopathic Intracranial Hypertension
Source: Eye (Lond). 2024 Jan 11;38(7):1374–9. doi: 10.1038/s41433-023-02908-y (PMC11076535; doi:10.1038/s41433-023-02908-y)
Supplement: Supplementary file 1 — Supplementary Material [file 41433_2023_2908_MOESM1_ESM.docx]

**Effect of Glucagon Like Peptide-1 receptor agonist exenatide, used as an intracranial pressure lowering agent, on cognition in Idiopathic Intracranial Hypertension.**

Olivia Grech^1,2^ James L Mitchell^1,2,3^ Hannah S Lyons^1,2,3^ Andreas Yiangou^1,2,3^ Mark Thaller^1,2,3^ Georgios Tsermoulas^1,2,4^ Kristian Brock^5^ Susan P Mollan^1,2,6^ Alexandra J Sinclair^1,2,3^

*1. Institute of Metabolism and Systems Research, College of Medical and Dental Sciences, University of Birmingham, Birmingham, B15 2TT, United Kingdom (UK).*

*2. Centre for Endocrinology, Diabetes and Metabolism, Birmingham Health Partners, Birmingham, B15 2TH, UK.*

*3. Department of Neurology, University Hospitals Birmingham NHS Foundation Trust, Queen Elizabeth Hospital, Birmingham, B15 2WB, UK.*

*4. Department of Neurosurgery, Queen Elizabeth Hospital Birmingham, University Hospitals of Birmingham, UK*

*5. Cancer Research Clinical Trials Unit, University of Birmingham, UK.*

*6. Birmingham Neuro-Ophthalmology, University Hospitals Birmingham NHS Foundation Trust, Queen Elizabeth Hospital, Birmingham, B15 2WB, UK.*

| **Inclusion Criteria** | **Exclusion Criteria** |
| --- | --- |
| - Female IIH patients aged between 18 and 60 years, diagnosed according to the revised diagnostic criteria for pseudotumour cerebri syndrome who have active disease (papilloedema [Frisen grade ≥ 1], significantly raised ICP >25cmH2O) and no evidence of venous sinus thrombosis (magnetic resonance imaging (MRI) or computerised tomography (CT) imaging and venography as noted at diagnosis). - Able to give informed consent. | - Age less than 18 or older than 60 years. - Pregnant or trying to conceive. - Significant co-morbidity; such that in the opinion of the investigator it would not be in the participant’s best interest to participate in the trial. - Addison’s or Cushing’s disease. - Previous CSF shunt, neurovascular stent or optic nerve sheath fenestration. - Currently using GLP-1 agonist or DPP-4 inhibitor. - Surgical contraindication. - Concomitant therapy with acetazolamide, topiramate or diuretics (this can be discontinued 1 month prior to enrolment). - Inability to give informed consent. |

**Supplementary Table 1** Inclusion and exclusion criteria.
